# Pus in the Blood; No Metastatic Abscesses

**Published:** 1863-07

**Authors:** 


PUS IN THE BLOOD; NO METASTATIC ABSCESSES.—Professor Langier has communicated to the *Gazette des Hopitaux* the case of a man, aged thirty-seven, who begged for amputation of the thigh on account of excruciating pain connected with white swelling of the knee. The patient died a week after the operation, having had several fits of shivering. M. Langier concluded, from the rapidity of the phenomena, that no metastatic abscesses would be found, but suspected that pus-globules might be discovered in the blood. M. Chatin examined three drachms of blood taken from the right side of the heart, and found pus-globules by the microscope in one of the three drachms. Ammonia gave a gelatinous precipitate in the second portion; and ammonia was evolved by the last drachm when left to decompose. From the first two results, M. Langier concludes that the blood contained pus.—*London Lancet.*

